# Isoflurane Post-Treatment Improves Outcome after an Embolic Stroke in Rabbits

**DOI:** 10.1371/journal.pone.0143931

**Published:** 2015-12-08

**Authors:** Feng Chen, Zonghong Long, Jinbo Yin, Zhiyi Zuo, Hong Li

**Affiliations:** 1 Department of Anesthesiology, Xinqiao Hospital, Third Military Medical University, Chongqing, China; 2 Department of Neurosurgery, Xinqiao Hospital, Third Military Medical University, Chongqing, China; 3 Department of Anesthesiology, University of Virginia, Charlottesville, Virginia, United States of America; University of Pennsylvania, UNITED STATES

## Abstract

Application of commonly used volatile anesthetics after brain ischemia onset (post-treatment) provides neuroprotection in rodents. To further test its translational potential, this study was designed to determine whether isoflurane post-treatment induced neuroprotection in rabbits after embolic stroke. White male New Zealand rabbits received intra-carotid injection of clots when they were awake. Some rabbits were exposed to 2.5% isoflurane for 1 h at 5 min after the injection. Isoflurane post-treatment increased the tolerance of rabbits to the amount of clots. Isoflurane post-treatment also reduced brain infarct volumes and plasma S100B 3 days after the injection of 5 mg clots and improved neurological deficit scores after the stroke. Isoflurane post-treatment improves neurological outcome in rabbits after embolic stroke.

## Introduction

Volatile anesthetics are the most commonly used general anesthetics in human in the U.S.A. Application of the currently clinically used volatile anesthetics, such as isoflurane and sevoflurane, after the onset of brain ischemia (post-treatment or post-conditioning) reduces ischemic brain injury [1–3]. These effects have been shown in rodents whose brain ischemia is induced by intravascular suture technique. Isoflurane post-treatment-induced neuroprotection has also been shown in rodents after hemorrhagic stroke [4]. To determine translational potential, it is necessary to test the neuroprotection of volatile anesthetic post-treatment in large animals. Also, ischemic stroke is often caused by emboli. Thus, we designed this study to determine whether isoflurane post-treatment induced neuroprotection in rabbits after an embolic stroke that is the model used to establish the effectiveness of tissue plasminogen activator therapy [5].

## Methods

Animal protocol was approved by the animal care and use committee of Third Military Medical University, Chongqing, China. Some animals would die due to stroke. This point was reviewed and approved by the committee. We did not use humane endpoints to determine when to euthanatize animals because mortality was a major outcome parameter in the study. All animal experiments were carried out in accordance with the National Institutes of Health Guide for the Care and Use of Laboratory Animals (NIH publications number 80–23) revised in 2011. Efforts were made to minimize the number of animals used and their suffering. Our manuscript was written up in accordance with the Animal Research: Reporting in vivo Experiments.

### Study groups of rabbits

White male New Zealand rabbits (2–3 months old) weighing 2 to 3 kg were randomly assigned to embolic stroke and embolic stroke plus isoflurane post-treatment groups. In the dose-response study, rabbits in the embolic stroke group received 2.5, 5, 7.5, 10, 12.5 and 15 mg blood clots, while rabbits in the embolic stroke plus isoflurane post-treatment group received 5, 7.5, 10, 12.5, 15 and 17.5 mg clots (n = 3 for each dose). In the second experiment, rabbits were randomly assigned to receive 5 mg clots for the embolic stroke and embolic stroke plus isoflurane post-treatment (n = 12 for each group). Total 17 rabbits died due to stroke before the end of 3-day observation time after the onset of stroke. All survived rabbits were euthanatized by carbon dioxide at the end of the observation time. During observation time, animals were assessed every hour for three times immediately after stroke and then every 8 h for the rest of time. Intensive care including frequent body turning and intravenous fluids was provided for those animals with stroke signs. In addition to the wound infiltration with bupivacaine, buprenorphine was given for those animals showing signs of pain.

### Emboli preparation

Blood was drawn from one rabbit and allowed to clot for 3 h at 37°C. Blood clot was then placed at 50°C for 10 h. They were crushed. The dried clots were filtered through a 240-um^2^ and then 100-um^2^ nylon net. The particles of clot were weighed, packed and sterilized by epoxy ethane.

### Embolic stroke and isoflurane post-treatment

Surgical procedures were performed as described previously [6]. Briefly, rabbits were anesthetized with isoflurane via a face mask, 5% in 3 L/min during induction and 2% in 3 L/min as a maintenance dose. Right external carotid artery and common carotid artery (CCA) were ligated. An arterial puncture needle was inserted into the right CCA distal to the ligation site. The catheter was left in place. The neck incision was closed and infiltrated with 0.25% bupivacaine but the distal end of the catheter was accessible outside. The catheter was filled with heparinized saline (25 units/mL) and plugged with an injection cap. Animals were recovered from anesthesia for 3 h and then received 1 mL sterile saline containing the intended amount of blood clots through the catheter. Rabbits were fully awake during the injection. Five minutes later, rabbits in the post-treatment group were treated with 2.5% isoflurane that was about 1.2 minimum alveolar concentrations for rabbits [7] in pure oxygen for 1 h. Those in embolic stroke group were exposed to 100% oxygen for 1 h.

### Neurological deficit scores

As described before [5], neurological function is scored blindly on a four-point scale by a person: 0) normal activity; 1) abnormal activity with any of the following: in circles, limb paralysis, head tilt or nystagmus; 2) more than two signs for score 1 or with seizure; 3) death [5, 8], Rabbits were evaluated 72 h after the stroke onset in the dose-response study or before the stroke, 5 min, 2 h and 72 h after the clot injection in the second experiment.

### Infarct volume assessment

Seventy two hours after the clot injection, rabbits were sacrificed by carbon dioxide. Brain was removed quickly and weighed, then cooled at -80°C for 10 min and sectioned into 2-mm thick coronal slices. Brain slices were incubated with 1% 2,3,5-triphenyltetrazolium chloride at 37°C for 30 min, fixed with 4% buffered formalin and stored overnight at 4°C. The stained sections were photographed to calculate infarct volume as we reported before [1].

### Water content analysis

Brain was dried by filter paper immediately after being removed from rabbits and weighed to obtain wet weight (W). After brain slices were used to calculate infarct volume, all slices from the brain were dried at 105°C for 24 h and weighed to obtain dry weight (D). The percentage of water content was calculated as (W-D)*100/W [4].

### ELISA assay of plasma S100B protein and neuron specific enolase (NSE)

Blood was collected before clot injection and 72 h after injection. Blood was centrifuged at 3000 r/min for 10 min at 4°C. Plasma was collected and store at -80°C. Plasma S100B and NSE concentrations were measured with kits from TSZ ELISA (Waltham, MA) and Cloud-Clone Corp (Houston, TX), respectively [8].

### Statistical analysis

Results are presented in mean ± S.D. Results were analyzed by t-test, two-way analysis of variance, two-way repeated measures analysis of variance as appropriate. A p ≤ 0.05 decision rule was utilized as the null hypothesis rejection criterion for all statistical comparisons.

## Results

**Fig 1:**
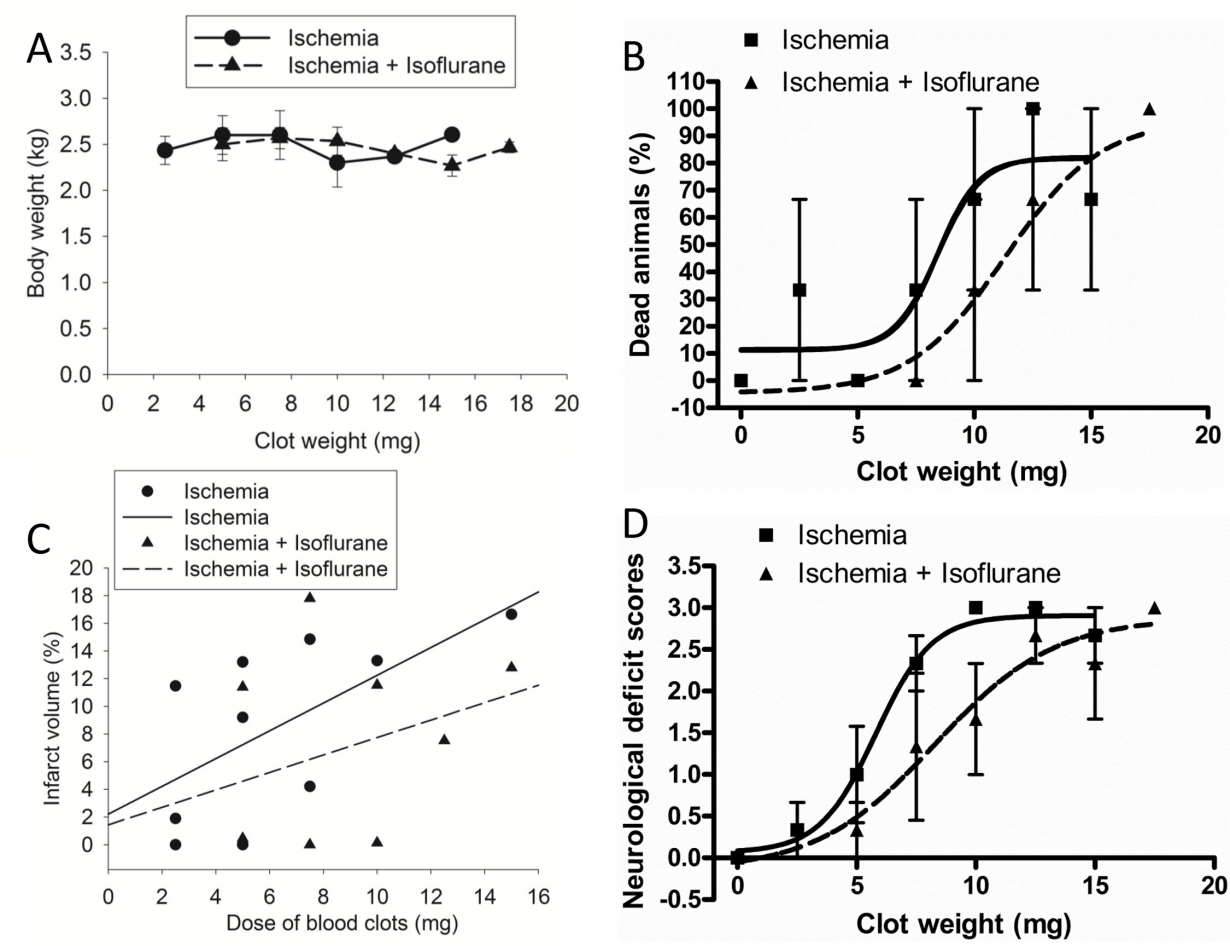
Dose-response. White male New Zealand rabbits were subjected to embolic stroke and post-treated with or without isoflurane. A: rabbit body weights, B: mortality, C: infarct volume, D: neurological deficit scores. Results were mean ± S.D. for panels A, B and D (n = 3 for each clot dose).

Data from all animals that completed the placement of the catheter in the right CCA were included in the analysis. The rabbits used for the clot dose-response study had similar body weights among various groups (Fig 1A). Embolic stroke caused significant mortality in rabbits. The EC50 for clots to cause death within the 72-h observation time was 8.4 ± 1.7 and 11.3 ± 2.1 mg (P < 0.001), respectively, for the embolic stroke group and embolic stroke plus isoflurane post-treatment group (Fig 1B). The infarct volume of the rabbits post-treated with isoflurane tended to be smaller than that of embolic stroke group (P = 0.2827 for comparison of the slopes of the best-fit lineal lines of animals with or without isoflurane post-treatment) (Fig 1C). This analysis only included the data from animals that survived till 72 h after the onset of embolic stroke. When neurological deficit scores of rabbits that survived or did not survive till 72 h after the onset of embolic stroke were pooled together, the clot dose to cause neurological deficit scores at 1.5 (half of the maximal neurological deficit scores) was 5.8 ± 0.6 and 8.3 ± 1.9 mg (P < 0.001), respectively, for the embolic stroke group and embolic stroke plus isoflurane post-treatment group (Fig 1D).

**Fig 2:**
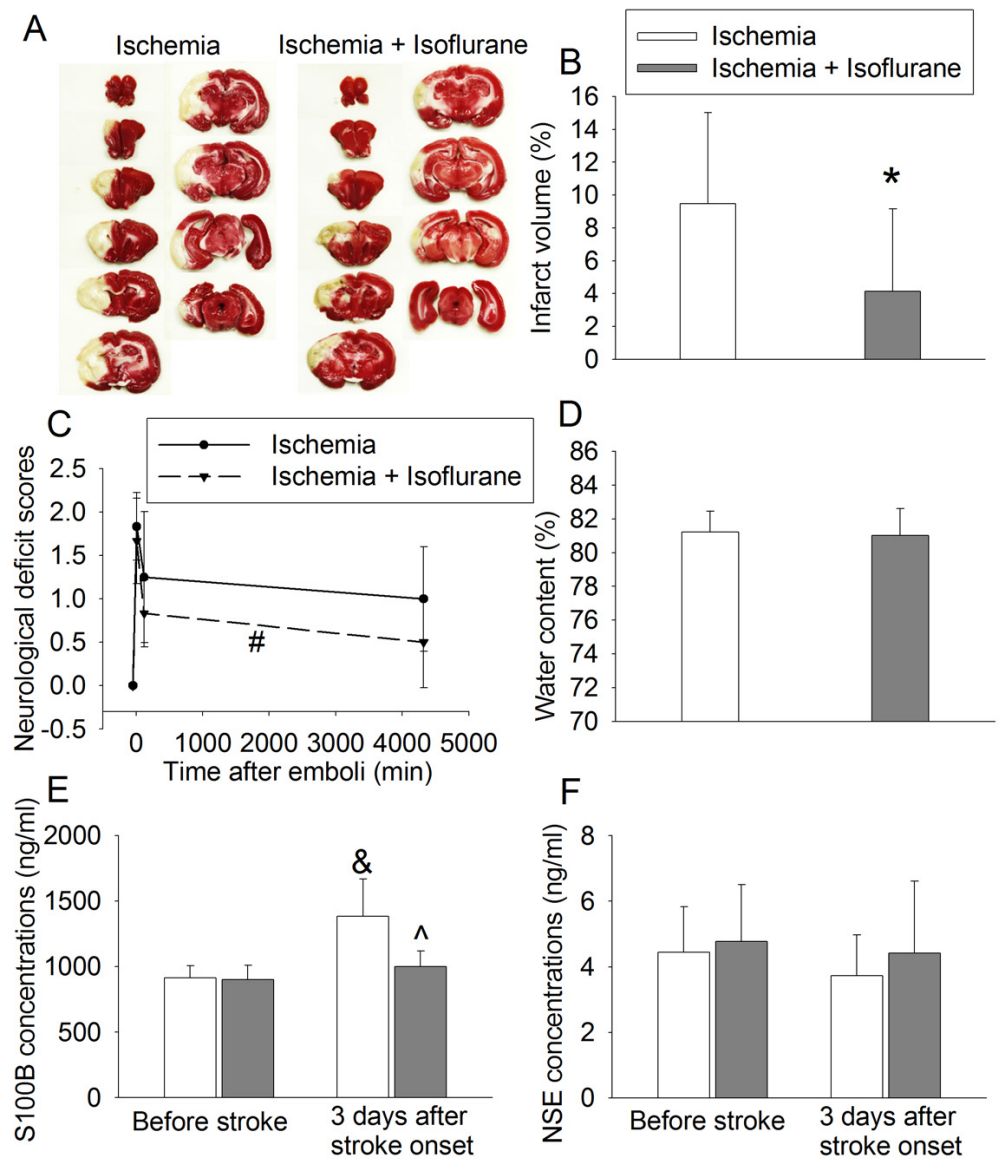
Isoflurane post-treatment-induced neuroprotection. White male New Zealand rabbits were subjected to embolic stroke induced by 5 mg clots and post-treated with or without isoflurane. A: representative images of brain slices after being stained with 2,3,5-triphenyltetrazolium chloride, B: infarct volume, C: neurological deficit scores, D: brain water content. Results were mean ± S.D. (n = 12). * P < 0.05 compared with ischemia group. # P < 0.05 compared with ischemia group by two-way repeated measures analysis of variance.

**Fig 3:**
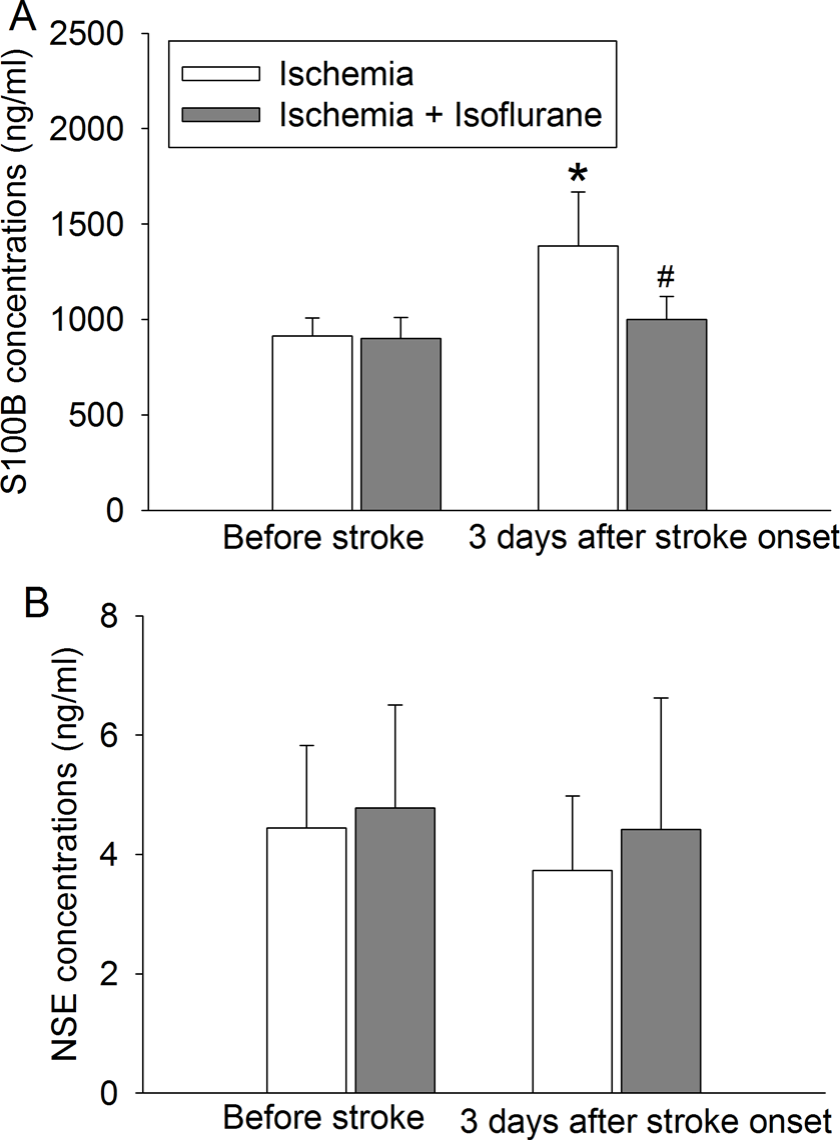
Effects of isoflurane post-treatment on S100B and NSE concentrations in the plasma. White male New Zealand rabbits were subjected to embolic stroke induced by 5 mg clots and post-treated with or without isoflurane. Blood was drawn before the stroke or 3 days after the stroke. A: S100B, B: NSE. Results were mean ± S.D. (n = 9). * P < 0.05 compared with the value before stroke in the same group. # P < 0.05 compared with the value of ischemia group at the same time.

To further test isoflurane post-treatment-induced neuroprotection, rabbits were subjected to 5 mg clots. This clot dose was chosen based on the results of our dose-response study to achieve minimal death but also produce significant brain infarction. The body weights of rabbits that had stroke alone or stroke plus isoflurane post-treatment were 2.6 ± 0.2 and 2.5 ± 0.2 kg (n = 12, P = 0.119), respectively. Rabbits post-treated with isoflurane had smaller infarct volumes than rabbits with stroke alone (Fig 2A and 2B). Isoflurane post-treatment also was a significant factor to affect neurological deficit scores after the stroke [F(1,22) = 4.452, P = 0.046] (Fig 2C). However, isoflurane post-treatment did not affect the amount of water in the brain (Fig 2D). The embolic stroke was a significant factor to increase S100B in the plasma [F(1, 32) = 25.333, P < 0.001] 3 days after the stroke. Isoflurane post-treatment significantly reduced this increase [F(1,32) = 12.441, P = 0.001] (Fig 3A). However, there were no significant changes in NSE 3 days after the stroke (Fig 3B).

## Discussion

Our results clearly suggest that isoflurane post-treatment provides neuroprotection in rabbits after an embolic stroke. This suggestion is supported by the findings that rabbits post-treated with isoflurane tolerated more clots than animals without post-treatment in the dose-response study and that isoflurane post-treatment reduced infarct brain volumes and neurological deficit scores in animals received intra-carotid injection of 5 mg clots. These findings represent initial evidence that isoflurane post-treatment provides neuroprotection in animals that are larger and higher ranking order than rodents. More importantly, a highly clinical relevant stroke model was used to test this neuroprotection. These results suggest the translational potential of isoflurane post-treatment in human; especially in the context that isoflurane has been used safely in clinical practice for decades.

Our results showed that embolic stroke increased plasma S100B and isoflurane post-treatment reduced this increase. This direction of changes is consistent with that of brain injury severity, which suggests that plasma S100B may be used to indicate brain injury. S100B is mainly expressed in the astrocytes [9]. Its increase in the blood has been shown in patients after ischemic stroke and is proposed as a marker for the blood-brain barrier injury that occurs in many diseases including stroke and brain trauma [10, 11]. NSE is mostly expressed in neurons and plasma NSE has been evaluated as a marker for brain injury [12]. Our results showed that plasma NSE was not increased 3 days after the stroke. Consistent with our finding, no change in the plasma NSE has been reported in patients with ischemic stroke, although its increase has also been shown [10].

We used 2.5% isoflurane in this study. One minimum alveolar concentration of isoflurane for rabbits is 2.05% [7]. Thus, we used about 1.2 minimum alveolar concentrations. This amount of anesthetics in the form of either one anesthetic or combination of multiple anesthetics is often used clinically to anesthetize some of our patients because 1 minimum alveolar concentration can only keep 50% subjects not to move in response to surgical stimulation and 1.2 minimum alveolar concentrations are needed to induce immobility on 95% subjects. The application of isoflurane in this study was 5 min after the onset of ischemic stroke. This design is because our previous study showed that isoflurane needed to be applied within 10 min after brain cell ischemia for it to be neuroprotective [13].

The previous study testing the effects of tissue plasminogen activator on neurological outcome after thrombotic stroke observed rabbits for 24 h and used mortality as an outcome parameter [5]. About 16 to 23% patients died in the acute phase of ischemic stroke [13, 14]. We used mortality as a parameter to reflect outcome in the dose-response study. We chose to observe rabbits for 3 days because stroke maturity may have occurred within 2 to 3 days after the onset of ischemic stroke [15].

Our study has limitations. First, we did not perform mechanistic study. Multiple mechanisms have been identified for isoflurane post-treatment-induced neuroprotection in rodents. These mechanisms include activating protective signaling and enhancing protective protein expression [3, 16]. We did not perform mechanistic studies here because our goal was to determine whether isoflurane post-treatment induced neuroprotection in large animals suffering from a clinically relevant stroke. Second, we used isoflurane during the procedure to implant intra-carotid catheter. This can induce preconditioning effect. However, no anesthetic is known clearly without this effect or does not have detrimental effects. Volatile anesthetics can be eliminated very quickly. Also, rabbits in both embolic stroke and embolic stroke plus isoflurane post-treatment groups had isoflurane exposure during the procedure. Thus, the improved neurological outcome in the embolic stroke plus isoflurane post-treatment group should be from isoflurane post-treatment.

Our finding has significant implication. Unlike preconditioning/pretreatment, post-treatment does not require the predication of occurrence of stroke for its application. Isoflurane post-treatment was provided very soon after the onset of embolic stroke in our study. Clinical situations where post-treatment can be provided very quickly do occur, for example, during surgery or interventional procedures.

In summary, our results suggest that isoflurane post-treatment is neuroprotective in rabbits after embolic stroke.
